# Neural Abnormalities in Bipolar Disorder: A Meta-Analysis of Functional Neuroimaging Studies

**DOI:** 10.1192/j.eurpsy.2024.914

**Published:** 2024-08-27

**Authors:** S. J. Herrera, F. A. Reyes, G. Johnson-Venegas, C. Baten, G. Zamora, A. M. Klassen, J. A. Miller, E. Woo, D. W. Hedges, P. J. Hamilton, I. H. Gotlib, M. D. Sacchet, C. H. Miller

**Affiliations:** ^1^Department of Psychology, California State University, Fresno, Fresno; ^2^Department of Psychology, Palo Alto University, Palo Alto; ^3^Department of Psychology, Brigham Young University, Provo, United States; ^4^Department of Biological and Medical Psychology, University of Bergen, Bergen, Norway; ^5^Department of Psychology, Stanford University, Palo Alto; ^6^Department of Psychiatry, Massachusetts General Hospital, Harvard Medical School, Boston, United States

## Abstract

**Introduction:**

Bipolar I disorder (BD-I) is a chronic and recurrent mood disorder characterized by alternating episodes of depression and mania; it is also associated with substantial morbidity and mortality and with clinically significant functional impairments. While previous studies have used functional magnetic resonance imaging (fMRI) to examine neural abnormalities associated with BD-I, they have yielded mixed findings, perhaps due to differences in sampling and experimental design, including highly variable mood states at the time of scan.

**Objectives:**

The purpose of this study is to advance our understanding of the neural basis of BD-I and mania, as measured by fMRI activation studies, and to inform the development of more effective brain-based diagnostic systems and clinical treatments.

**Methods:**

We conducted a large-scale meta-analysis of whole-brain fMRI activation studies that compared participants with BD-I, assessed during a manic episode, to age-matched healthy controls. Following PRISMA guidelines, we conducted a comprehensive PubMed literature search using two independent coding teams to evaluate primary studies according to pre-established inclusion criteria. We then used multilevel kernel density analysis (MKDA), a well-established, voxel-wise, whole-brain, meta-analytic approach, to quantitatively synthesize all qualifying primary fMRI activation studies of mania. We used ensemble thresholding (p<0.05-0.0001) to minimize cluster size detection bias, and 10,000 Monte Carlo simulations to correct for multiple comparisons.

**Results:**

We found that participants with BD-I (N=2,042), during an active episode of mania and relative to age-matched healthy controls (N=1,764), exhibit a pattern of significantly (p<0.05-0.0001; FWE-corrected) different activation in multiple brain regions of the cerebral cortex and basal ganglia across a variety of experimental tasks.

**Conclusions:**

This study supports the formulation of a robust neural basis for BD-I during manic episodes and advances our understanding of the pattern of abnormal activation in this disorder. These results may inform the development of novel brain-based clinical tools for bipolar disorder such as diagnostic biomarkers, non-invasive brain stimulation, and treatment-matching protocols. Future studies should compare the neural signatures of BD-I to other related disorders to facilitate the development of protocols for differential diagnosis and improve treatment outcomes in patients with BD-I.

**Disclosure of Interest:**

None Declared

